# Outcome of Holiday and Nonholiday Admission Patients with Acute Peptic Ulcer Bleeding: A Real-World Report from Southern Taiwan

**DOI:** 10.1155/2014/906531

**Published:** 2014-07-01

**Authors:** Tsung-Chin Wu, Seng-Kee Chuah, Kuo-Chin Chang, Cheng-Kun Wu, Chung-Huang Kuo, Keng-Liang Wu, Yi-Chun Chiu, Tsung-Hui Hu, Wei-Chen Tai

**Affiliations:** Division of Hepatogastroenterology, Department of Internal Medicine, Kaohsiung Chang Gung Memorial Hospital and Chang Gung University College of Medicine, 123 Ta-Pei Road, Niaosung Hsiang, Kaohsiung City 833, Taiwan

## Abstract

*Background*. Recent findings suggest that patients admitted on the weekend with peptic ulcer bleeding might be at increased risk of adverse outcomes. However, other reports found that there was no “holiday effect.” The purpose of this study was to determine if these findings hold true for a real-life Taiwanese medical gastroenterology practice. *Materials and Methods*. We reviewed the medical files of hospital admissions for patients with peptic ulcer bleeding who received initial endoscopic hemostasis between January 2009 and March 2011. A total of 744 patients were enrolled (nonholiday group, *n* = 615; holiday group, *n* = 129) after applying strict exclusion criteria. Holidays were defined as weekends and national holidays in Taiwan. *Results*. Our results showed that there was no significant difference in baseline characteristics between the two groups. We also observed that, compared to the nonholiday group, patients in the holiday group received earlier endoscopy treatment (12.20 hours versus 16.68 hours, *P* = 0.005), needed less transfused blood (4.8 units versus 6.6 units, *P* = 0.02), shifted from intravenous to oral proton-pump inhibitors (PPIs) more quickly (5.3 days versus 6.9 days, *P* = 0.05), and had shorter hospital stays (13.05 days versus 17.36 days, *P* = 0.005). In the holiday and nonholiday groups, the rebleeding rates were 17.8% and 23.41% (*P* = 0.167), the mortality rates were 11.63% versus 13.66% (*P* = 0.537), and surgery was required in 2.11% versus 4.66% (*P* = 0.093), respectively. *Conclusions*. Patients who presented with peptic ulcer bleeding on holidays did not experience delayed endoscopy or increased adverse outcomes. In fact, patients who received endoscopic hemostasis on the holiday had shorter waiting times, needed less transfused blood, switched to oral PPIs quicker, and experienced shorter hospital stays.

## 1. Introduction 

Peptic ulcer bleeding is a common cause of hospitalization, and mortality remains at 6–8% despite advances in both pharmacologic and endoscopic therapies [1, 2]. Reports regarding outcomes for different management regimens for peptic ulcer bleeding patients during holidays are inconsistent. Some described increased adverse outcomes on holidays [3, 4] while others did not [5, 6].

It is well documented that the risk for recurrent bleeding is increased in patients with high-risk peptic ulcers after initial endoscopic hemostasis, although it can control bleeding and reduce the rebleeding, morbidity, and mortality rates [7, 8]. Theoretically, the possibility of greater risk on holidays is due to potential lower staffing levels, less senior staff, cross-cover of clinical specialties, and a lower likelihood that invasive procedures, such as endoscopy, will be performed on holidays. Peptic ulcer bleeding is a common medical emergency in Taiwan that challenges both gastroenterologists and general surgeons. The purpose of this study was to determine whether the holiday effect occurred in our hospital. We analyzed the outcomes of patients with peptic ulcer bleeding who presented on holidays compared to those admitted on nonholidays. The endpoints were rebleeding, need for surgery, and mortality.

## 2. Materials and Methods

### 2.1. Study Design

**Figure 1:**
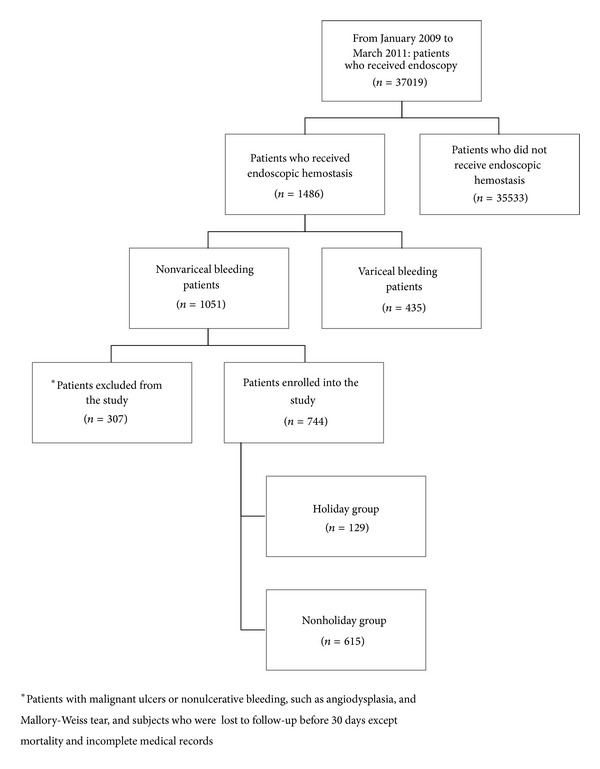
Schematic flowchart of the study design and the patient numbers during follow-up.

Between January 2009 and March 2011, we performed 37,019 esophagogastroduodenoscopy studies in our endoscopic center. Among them, 1,051 patients underwent endoscopic hemostasis for confirmed gastric and duodenal ulcer bleeding. All subjects received the intravenous proton-pump inhibitors (PPIs). After the medical records of these 1,051 patients were reviewed, we excluded 307 patients with malignant ulcers and nonulcerative bleeding (e.g., angiodysplasia, Mallory-Weiss tear), subjects lost to follow-up before 30 days (except those who died), and patients with incomplete chart records. Eventually, we included 744 patients in this study. We divided these patients into holiday (*n* = 129) and nonholiday groups (*n* = 615) (Figure 1). Gastric or duodenal ulcers bleeding was diagnosed by the gastroscopy (GIF-Q260; Olympus Optical Co., Ltd., Tokyo, Japan) and clinical signs of hematemesis, coffee ground vomitus, hematochezia, or melena. The time from admission to endoscopic treatment was measured, and the bleeding source was identified. Patients' statuses were stratified according to the Rockall score system [9]. All of our patients received endoscopic interventions within 24 hours of arriving at the emergency room, and endoscopic hemostasis interventions were performed by experienced endoscopists. In our hospital, our endoscopic center provides therapeutic endoscopic services 24 hours a day. The registered clinical variables were demographic data; clinical manifestations of bleeding; time to endoscopy; the use of tobacco, alcohol, aspirin, clopidogrel, and nonsteroidal anti-inflammatory drugs (NSAIDs); and comorbidities such as diabetes mellitus, cardiovascular disease, stroke, chronic kidney disease (CKD), and chronic obstructive pulmonary disease. Other clinical characteristics, such as age, sex, and hemodynamic instability on admission, and laboratory data, including hemoglobin, platelet count, and international normalized ratio, were analyzed. The endpoints were rebleeding, need for surgery, and mortality.

This retrospective chart review study was approved by both the Institutional Review Board and the Ethics Committee of Chang Gung Memorial Hospital, Taiwan (IRB103-1639B). All patients were at least 18 years old and provided written informed consent before undergoing endoscopic interventions.

### 2.2. Definitions

The holidays were defined as all national holidays and weekends in Taiwan during the study period. Patients with peptic ulcer bleeding were treated with intravenous high-dose PPIs (pantoprazole or esomeprazole 80 mg bolus followed by 200 mg continuous infusion for 3 days). Rebleeding was defined as a new onset of hematemesis, melena, fresh blood or coffee ground material in the nasogastric (NG) tube, or both associated with tachycardia or hypovolemic shock or a decrease in serum hemoglobin level >2 g/dL after successful endoscopic and pharmacological treatment, and hemodynamic stability of at least a 24-hour period of stable vital signs [10–12]. Bleeding recurrence was confirmed by endoscopy in all cases. Shock was defined as tachycardia, heart rate ≥ 100/min, or hypotension (systemic blood pressure ≤ 90 mmHg) [13–16].

### 2.3. Endoscopic Assessment

Endoscopic signs of high-risk ulcers were defined according to the Forrest classification [16]. In high-risk stigmata, active bleeding was defined as continuous blood spurting (Forrest IA) or oozing (Forrest IB) from the ulcer base. A nonbleeding vessel visible at endoscopy was defined as a discrete protuberance at the ulcer base (Forrest IIA). An adherent clot was resistant to forceful irrigation or suction (Forrest IIB). In low-risk stigmata, flat, pigmented spots or clean bases were defined as Forrest grade IIC or III. We performed endoscopic hemostasis for all patients with peptic ulcers and high-risk stigmata.

### 2.4. Statistical Analysis

The Statistical Package for Social Sciences (SPSS22.0 for Windows, IBM Corp., Armonk, NY, USA) was used to analyze the data. The results are expressed as distributions, absolute frequencies, relative frequencies, medians and ranges, or mean ± SD. The quantitative data were compared using Student's *t*-test for normally distributed variables. Differences between the proportions of categorical data were evaluated with the *χ* ^2^ test or with Fisher's exact test when the number of expected subjects was less than five. Differences were considered statistically significant at *P* < 0.05.

## 3. Results 

### 3.1. Demographic and Clinical Characteristics

The patients' demographics and clinical characteristics are shown in Table 1. There were no significant differences between the two groups.

### 3.2. Rebleeding and Receipt of Red Cell Transfusion

**Figure 2:**
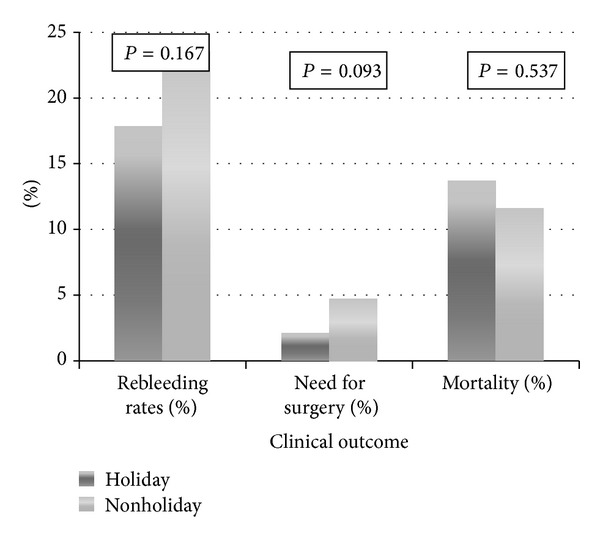
The clinical outcomes of holiday and nonholiday patients.

Univariate analysis revealed that the percentages of patients who experienced rebleeding were comparable between the nonholiday and holiday groups (23.4% versus 17.8%, *P* = 0.167; Table 2). The holiday group required smaller amounts of transfused blood (4.8 ± 5.2 units versus 6.6 ± 9.3 units, *P* = 0.02; Figure 2).

### 3.3. Time to Endoscopy, Length of Hospital Stay, and Time to Oral PPI

We found that patients in the holiday group received earlier endoscopy treatment (12.2 ± 15.3 h versus 16.7 ± 19.8 h, *P* = 0.008; Table 2). In addition, patients in the nonholiday group required longer hospital stays than the holiday group (17.4 ± 28.2 days versus 12.1 ± 12.5 days, *P* = 0.005) and required more time to shift from intravenous to oral PPI (6.9 ± 9.1 days versus 5.3 ± 6.1 days, *P* = 0.05; Table 2).

### 3.4. Surgical Intervention and Mortality

There was no significant difference in the number of patients who required surgeries between the two groups, but a trend toward significance was observed for the nonholiday group patients and higher rate of surgical intervention (2.1% versus 4.7%, *P* = 0.093; Figure 2). There was no significant difference in mortality between the two groups (13.66% versus 11.63%, *P* = 0.537; Figure 2).

## 4. Discussion

A growing body of health services research indicates that increased mortality is associated with admission to hospitals on the weekends [17–20]. This issue has raised concern over the quality care of very important medical and surgical emergencies, including peptic ulcer bleeding, on holidays. The existing reports reached inconsistent results. Some studies describe increased rates of adverse outcomes [3, 4, 21], whereas some reported that there is no evidence of a “holiday effect” [5, 6, 22]. The present study suggests that the holiday effect was not observed for patients with peptic ulcer bleeding who were treated in our hospital. The percentages of patients who suffered from rebleeding and mortality and those who needed surgery were comparable between the nonholiday and holiday patients. In fact, the holiday group required less transfused blood, had a shorter time to endoscopy, more quickly shifted from intravenous to oral PPI, and had shorter hospital stays.

Generally, the outcome of treatment for peptic ulcer bleeding should be much improved given the emergence of more potent medications such as PPIs, increased use of dual endoscopic therapy and endoscopic triage for risk stratification, and advances in general medical care. Shaheen et al. observed an overall 25% reduction in the odds of mortality, irrespective of the day of admission, when comparing the 2000–2005 and 1993–1999 time periods [21]. However, patients admitted to hospital on the weekend for peptic ulcer-related hemorrhage have a higher mortality rate and more frequently undergo surgery [4]. For those reports suggesting a weekend or holiday effect of peptic ulcer bleeding, the explanations included lower staffing levels, less senior staff, cross-cover of clinical specialties, and a lower likelihood that invasive procedures, such as endoscopy, will be undertaken on weekends [4, 21]. Early endoscopic interventions and the first 72 hours of hospitalization are crucial for favorable outcomes in patients with peptic ulcer bleeding, especially the rebleeding rates. Therefore, the availability of early access to upper endoscopy and physicians with endoscopic expertise are key factors to treatment success [23–25]. A lack of staff on holidays and subsequent delays in upper endoscopy may explain the poorer outcomes. However, Ananthakrishnan et al. observed that the difference in mortality for weekend admissions was only significant among patients who did not undergo endoscopic intervention, with similar outcomes among the groups that did undergo emergency endoscopy [4]. Large population-based reports from the US, Canada, and the Netherlands describe the rates of early endoscopy as 72%, 76%, and 78%, respectively [26–28], but these data were from older publications. Recently, Haas et al. reported that >94% of patients with peptic ulcer bleeding undergo upper endoscopy within 24 hours. In the current study, 83.2% of patients with peptic ulcer bleeding underwent upper endoscopy within 24 hours.

Recent publications have suggested that the holiday effect did not influence the outcome of peptic ulcer bleeding [6, 22], which is in accordance with the present results. Those studies that did not find differences were probably performed in hospitals that were able to provide full-time therapeutic endoscopic services for early endoscopy. Therefore, the effects of lower staffing levels, less senior staff, and lower likelihood of invasive procedure on holidays were not problematic in these hospitals. Indeed, we found that patients in the holiday group received earlier endoscopy treatment than nonholiday patients (12.2 ± 15.3 h versus 16.7 ± 19.8 h, *P* = 0.008).

Nevertheless, previous studies also pointed out that patients presenting on weekends might be more critically ill than those presenting on weekdays. However, we observed that patients admitted with peptic ulcer bleeding on holidays and nonholidays were comparable for coexisting illness, shock status, and Rockall scores. Nahon et al. reported similar findings in a post hoc subanalysis of a prospective study performed in 53 general nonuniversity hospitals in France [22]. They further explained that the severity of bleeding estimated by the Rockall score and the rates of endoscopic interventions for active bleeding did not differ between weekend and weekday admissions. Importantly, a senior gastrointestinal specialist was on call and available on weekends in their hospitals. In addition, we had well-trained emergency room physicians who understood and adhered to the guidelines for early risk stratification in patients with peptic ulcer bleeding [23, 24].

In Taiwan, medical care is well covered by the National Health Insurance system. Patients are allowed to seek medical care in referral hospitals, including medical centers and university hospitals, regardless of the severity of their illness. For instance, patients with peptic ulcer bleeding are allowed to go to a hospital center with full-time therapeutic endoscopic services. In our endoscopic teams, a senior gastroenterology specialist who supervises the lower level staff members is always on call, even on holidays. This is necessary to minimize the difference of endoscopist skill and reduce waiting time, regardless of whether it is a holiday or nonholiday. This could also be one reason why the patients with peptic ulcer bleeding admitted on holidays achieved good outcomes in the present study.

This study was limited by the fact that it was a retrospective chart review study performed at a single institution, which could have resulted in sampling bias. In conclusion, patients who present with peptic ulcer bleeding on holidays did not experience delayed endoscopy or increased adverse outcomes, such as recurrent bleeding or mortality. In fact, patients who received endoscopic hemostasis on holidays experienced shorter waiting times, required less transfused blood, were more quickly shifted from intravenous to oral PPI, and had shorter hospital stays.

## Figures and Tables

**Table 1:** Baseline characteristics of nonholiday and holiday groups.

Characteristics	Nonholiday group (*n* = 615)	Holiday group (*n* = 129)	*P* value
Age (yr)	64.6 ± 14.1	66.45 ± 14.1	0.978
Female gender, *n* (%)	195 (32%)	48 (37%)	0.226
Hb (g/dL)	9.3 ± 2.8	9.2 ± 2.7	0.848
Platelets (×10^3^/*μ*L)	190.1 ± 99.3	205.4 ± 120.1	0.244
INR	1.24 ± 0.64	1.17 ± 0.49	0.116
Use of NSAIDs, *n* (%)	72 (12%)	9 (7%)	0.117
Use of aspirin, *n* (%)	93 (15%)	18 (14%)	0.735
Use of clopidogrel, *n* (%)	65 (11%)	14 (11%)	0.924
Use of warfarin, *n* (%)	32 (5%)	5 (4%)	0.528
Shock at presentation	311 (51%)	76 (59%)	0.084
Coexisting illness, *n* (%)			
CKD III, IV/V	204/83 (33%/13%)	49/11 (40%/9%)	0.245
COPD	44 (7%)	11 (9%)	0.588
CAD	110 (18%)	19 (15%)	0.389
DM	199 (32%)	36 (28%)	0.323
CVA	105 (17%)	24 (19%)	0.676
HTN	326 (53%)	63 (49%)	0.389
Cancer	116 (19%)	24 (19%)	0.946
Liver cirrhosis	115 (19%)	20 (16%)	0.392
Rockall score	6.2 ± 1.7	6.0 ± 1.8	0.727
Ulcer size (cm)	1.1 ± 0.7	1.2 ± 0.8	0.434
Forrest classification			
Ia/Ib/IIa/IIb/IIc/III	44/348/67/140/14/2	8/62/20/33/6/0	0.260
High stigmata, *n* (%)	599 (97.3%)	123 (95.3%)	0.212

Hb: hemoglobin; NSAIDs: nonsteroidal anti-inflammatory drugs; CKD: chronic kidney disease; COPD: chronic obstructive pulmonary disease; CAD: coronary artery disease; DM: diabetes mellitus; CVA: cerebral vascular accident; INR: international normalized ratio HTN: hypertension.

**Table 2:** Clinical outcomes for all patients presenting with acute upper gastrointestinal bleeding.

Characteristics	Nonholiday group (*n* = 615)	Holiday group (*n* = 129)	*P* value
Time to oral PPI (days)	6.9 ± 9.1	5.3 ± 6.1	0.05∗
Rebleeding, *n* (%)	144 (23.4%)	23 (17.8%)	0.167
Surgery, *n* (%)	13 (2.1%)	6 (4.7%)	0.097
Hospital stay (days)	17.4 ± 28.2	12.1 ± 12.5	0.005∗
Mortality, *n* (%)	84 (13.7%)	15 (11.6%)	0.776
Bleeding related/other causes	24 (3.9%)/60 (9.8%)	5 (3.9%)/10 (7.7%)	
Time to endoscopy (h)	16.7 ± 19.8	12.2 ± 15.3	0.008∗
PRBC BT (U)	6.6 ± 9.3	4.8 ± 5.2	0.020∗

*A significant value.

PRBC BT: blood transfusion of packed red blood cell; PPI: proton pump inhibitor.
